# Ultrathin Hematite Photoanode with Gradient Ti Doping

**DOI:** 10.34133/2020/5473217

**Published:** 2020-02-24

**Authors:** Pengfei Liu, Chongwu Wang, Lijie Wang, Xuefeng Wu, Lirong Zheng, Hua Gui Yang

**Affiliations:** ^1^Key Laboratory for Ultrafine Materials of Ministry of Education, Shanghai Engineering Research Center of Hierarchical Nanomaterials, School of Materials Science and Engineering, East China University of Science and Technology, 130 Meilong Road, Shanghai 200237, China; ^2^School of Electrical and Electronic Engineering, Nanyang Technological University, 50 Nanyang Avenue, Singapore, Singapore 639798; ^3^Institute of High Energy Physics, Chinese Academy of Sciences, Beijing 100049, China

## Abstract

The poor photoelectrochemical (PEC) performance derived from insufficient charge separation in hematite photoanode crucially limits its application. Gradient doping with band bending in a large region is then considered as a promising strategy, facilitating the charge transfer ability due to the built-in electric field. Herein, we developed a synthetic strategy to prepare gradient Ti-doped ultrathin hematite photoelectrode and systematically investigated its PEC performance. The as-synthesized electrode (1.5-6.0% doping level from the surface to the substrate) delivered a photocurrent of about 1.30 mA cm^−2^ at 1.23 V versus the reversible hydrogen electrode (RHE), which is nearly 100% higher than that of homogeneously doped hematite electrode. The enhanced charge transfer property, induced by the energy band bending due to the built-in electric field, has been further confirmed by electrochemical measurements. This strategy of gradient doping should be adaptable and can be applied for other functional materials in various fields.

## 1. Introduction

Solar-to-hydrogen production is a promising approach to convert intermittent solar energy into sustainable and green solar fuels (e.g., hydrogen), among which semiconductor-based photoelectrochemical (PEC) water splitting has attracted intensive research [1–3]. Over the last 40 years, numerous semiconductors (*α*-Fe_2_O_3_, WO_3_, BiVO_4_, Ta_3_N_5_, Si, Cu_2_O, InP, and others) have been investigated as photoelectrodes for PEC water splitting [1, 4–11], and hematite emerges as a promising candidate due to its intrinsic properties, including chemical stability, earth abundance, and a 2.1 eV bandgap that absorbs approximately 40% of the solar spectrum [4, 8, 12–15]. Unfortunately, although featuring a potential to convert 16.8% of solar energy to hydrogen, the practical application of hematite photoanode is crucially limited by its poor charge transportation, which further leads to significant charge recombination. To boost the charge transfer ability for further improving PEC performance, various strategies have been recently proposed, such as element doping, nanostructuring, and surface functionalization [4, 16–20].

Gradient doping is an efficient strategy of facilitating the charge transfer due to the resulting built-in electric field and has been thoroughly investigated in many fields, including photocatalysis, photovoltaics, and PEC water splitting [21–25]. For example, Abdi and coworkers developed gradient W-doped BiVO_4_ photoanodes *via* the layer-by-layer spray pyrolysis method, consisting of 10-step different gradient W concentration, which ultimately facilitated the efficiency of photogenerated carriers because of the built-in electric field [23]. However, the strategy of such tactfully introducing gradient in doping profile has been rarely conducted for hematite-based photoanodes, and it mainly focuses on surface-doped hematite with nonmetallic element P, which might not be the ideal choice for hematite photoelectrode [24, 26]. Moreover, the surface doping only functionalizes near the semiconductor/liquid interface andcannot facilitate the charge transfer in the bulk, which significantly limits the overall PEC water splitting performance [18].

**Figure 1:**
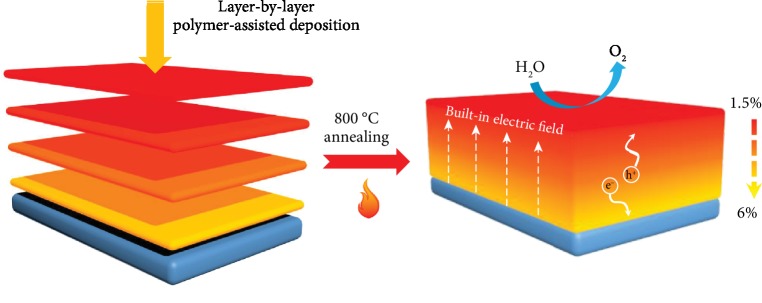
Schematic illustration of the PAD synthesized gradient-doped hematite photoanode. The built-in electric field will facilitate the charge transfer.

Herein, we synthesized ultrathin hematite photoanode *via* a layer-by-layer polymer-assisted deposition (PAD) method. Each layer of hematite is around 30 nm in thickness with different Ti-doping level, as shown in Figure 1. The built-in electric field, due to the gradient Ti concentration to distribute the band bending over the bulk of the hematite photoanode, then facilitates the separation of light-induced charges, contributing to the much enhanced photocurrent of water oxidation for the gradient-doped electrode (1.5-6.0% doping level from the surface to the substrate).

## 2. Results

### 2.1. Morphology Analysis

**Figure 2:**
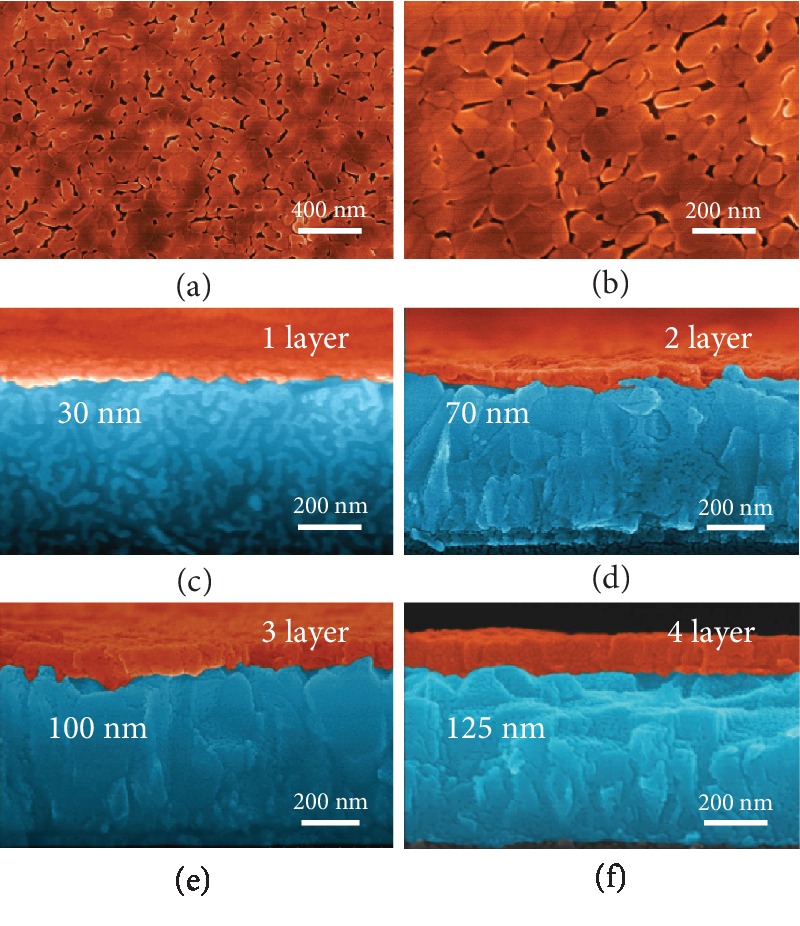
(a, b) SEM images of the surface of the PAD hematite film. The PAD film is composed of hematite nanoparticles with 30-60 nm in size. (c–f) Cross-section-viewed images of hematite electrode composed of 1-4-layered PAD films. The thicknesses are 30, 70, 100, and 125 nm, respectively.

To obtain the gradient Ti-doped Fe_2_O_3_ films, a multistep layer-by-layer PAD strategy was conducted with each step precisely controlling the weight concentration of Ti dopants (see more details in Materials and Methods). The typical morphologies of the PAD films were investigated by scanning electron microscopy (SEM) measurements. In Figure 2(a), the top-viewed SEM image shows that the PAD films (4 layers) are relatively flat and compact because the deposition precursors are composed of ethylenediaminetetraacetic acid (EDTA) coordinated cations (Fe or Ti) cross-linked by polyethylenimine (PEI) long chains *via* ultrafiltering the excessive uncoordinated metal cations or complex, which might individually nucleate and affect the quality of the films [27]. The high-resolution SEM image (Figure 2(b)) exhibits that the films consist of nanoparticles with an average size ranging from 30 to 60 nm. It is mentionable that the charge carrier diffusion length is about 10 nm and the particle size is appropriate for charge separation and transfer onto the surface to motivate oxygen generation [13]. Figures 2(c)–2(f) showcase the cross-section-viewed SEM images of PAD Fe_2_O_3_ films with different deposition layers. The films exhibit different thicknesses of 30, 70, 100, and 125 nm for 1-4-layered hematite films, respectively, with average film thickness of about 30 nm. Notably, the suitable thickness of hematite should be around 120 nm to guarantee the sufficient light absorption, but too thick films would also affect the charge carrier transfer efficiency with charge recombination during the transfer process [4]. Therefore, the optimum deposition times are 4 times with film thickness of 125 nm in this work.

### 2.2. Composition Analysis and Structural Characterizations

**Figure 3:**
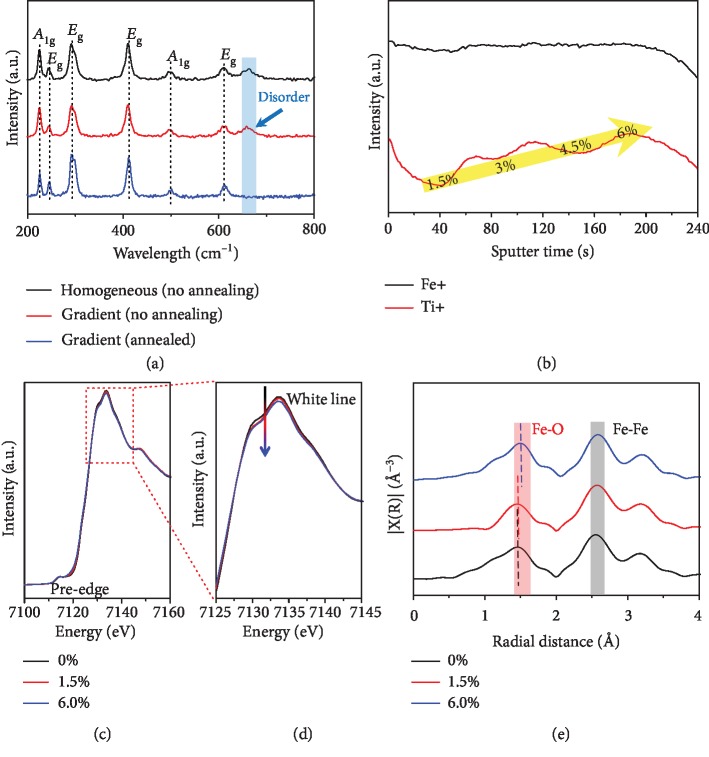
(a) Raman patterns of homogeneously doped (3.0%), gradient-doped (1.5-6.0%), and annealed gradient-doped (1.5-6.0%) PAD electrodes. The missing peak around 660 cm^−1^ for annealed film indicates that the annealing process at 800°C eliminates the doping-induced disorders. (b) TOF-SIMS spectrum of gradient hematite films, indicating the gradient distribution of Ti element. (c–e) XAFS spectra of hematite electrodes with different doping concentrations with (c, d) XANES spectra illustrating the change of valance states and local coordination of Fe element and (e) EXAFS spectra indicating the alteration of Fe-O bond length.

To further analyze the composition of the PAD films, we collected the samples of 4-layered homogenous films (3.0% Ti-doping level in each layer without annealing), gradient films (1.5-6.0% Ti-doping level from the surface to the substrate without annealing), and annealing-activated gradient films for X-ray diffraction (XRD) characterizations. In [Supplementary-material supplementary-material-1], the XRD patterns of different films all display no characteristic crystalline peaks besides the strong substrate signals of SnO_2_, which is ascribed to that the thickness of the hematite film which is too thin. To avoid the deficiency of XRD characterization in this work, we carried out the Raman analysis for the above samples. In Figure 3(a), the Raman spectra of all the three samples exhibit the typical bands of the hematite with *A*_1g_ located at 224 and 498 cm^−1^ as well as *E*_g_ located at 244, 291, 410, and 611 cm^−1^, demonstrating the crystal structure of the PAD films [13]. We note that the annealing activation treatment would diminish the characteristic band located around 660 cm^−1^, which might result from doping-induced disorder response signals [13]. As a result, the annealing treatment would also activate the pristine PAD gradient films with more ordered symmetric structures to avoid the charge recombination. It is notable that all the tested photoelectrodes were annealed if not mentioned.

To shed light on the surface valence states of Ti and Fe on the PAD films, we conducted the X-ray photoelectron spectroscopy (XPS) measurements on the typical surface 1.5% Ti-doped films. In [Supplementary-material supplementary-material-1], the Ti deconvolution XPS spectrum exhibits characteristic peaks centered at 457.9 and 463.7 eV for Ti 2p_3/2_ and 2p_1/2_ regions, respectively. We note that the narrow single feature of Ti 2p_3/2_ locates at 457.9 eV, lying between Ti^4+^ and Ti^3+^ in TiO_2_ (458.8 and 457.1 eV, respectively), which corresponds to the Ti^4+^ species included in a hematite host matrix [28]. While for the Fe elements, the fitted peaks located at 711.0 eV with satellite peaks at 719.3 eV in the Fe 2p_3/2_ regions are ascribed to Fe^3+^ components, and no obvious characteristic peak centered at 717.2 eV of Fe^2+^ has been detected, which further unambiguously demonstrates the main existence of Fe^3+^ in the gradient films [29].

Time-of-flight secondary ion mass spectrometry (ToF-SIMS) with high surface sensitivity is always used for unravelling the spatial distribution of chemical components [22]. In this work, we carried out the ToF-SIMS characterizations to identify the gradient Ti doping. In Figure 3(b), the signals of Fe ions nearly keep the same with the prolonging of the sputter time, which indicates the homogeneous distribution of Fe^3+^ in the films. For comparison, the signals of Ti ions gradually increase, corresponding to the gradient distribution of Ti dopants in each layers, which distinctly demonstrates the successful manipulation of gradient Ti doping in the PAD Fe_2_O_3_ films.

X-ray absorption fine structure (XAFS) analysis was also conducted to further investigate the local structures of the doped PAD films. We collected the PAD films with different Ti-doping levels (1 layer, named 0%, 1.5%, and 6.0%) for characterization. In Figure 3(c) of the X-ray absorption near edge structure (XAENS) of Fe K-edge spectra, all the three samples exhibit the same bulk valence states of Fe^3+^. In addition, the pre-edge centered at 7114 eV originates from the quadrupole 1 s⟶3 d transition, and no obvious difference has been observed for these samples. However, we found that the intensities of the white line peaks decreased with the rising Ti-doping levels, suggesting a lowering of the coordination number (CN) of Fe (Fe-O) when introducing Ti heteroatoms (Figure 3(d)). The enlarged XAENS spectra shown in [Supplementary-material supplementary-material-1] indicate the slightly decreased oxidation states due to Ti^4+^ dopant-substituted Fe^3+^ ions in the hematite films. We further studied the Fourier-transformed spectra of the extended X-ray absorption fine structure (EXAFS) spectroscopy of different doping-level samples for Fe K-edge. In Figure 3(e), it is obvious that the Fe-O length for 6.0% Ti^4+^ doped Fe_2_O_3_ located at 1.50 Å (without phase correction) is longer than that of 1.5% (1.46 Å) and 0% (1.45 Å) doped films, which also indicates the high-content Ti^4+^ doping might destroy the symmetrical structure of octahedral centers. All in all, on the basis of the XAFS results, we speculate that the high-doping level of 6.0% Ti^4+^-substituted Fe^3+^ would trigger the disordered structures on the surface, which might induce the charge recombination and further degrade the PEC performances.

### 2.3. Photochemical Water Oxidation Performance Evaluation

To evaluate the PEC water oxidation performance, all the PAD hematite photoanodes were tested in a 1.0 M NaOH electrolyte (pH = 13.6) applied with linear sweep bias voltage to obtain the photocurrent density. Firstly, to determine the optimum deposition layers of the PAD hematite, we fabricated the PAD film controls without any Ti dopants from 1-5 layers. As shown in [Supplementary-material supplementary-material-1], the linear sweep voltammetric (LSV) curves exhibit that the photocurrent density gradually increases from 1 to 4 layers (without doping), with the highest photocurrent density of 0.18 mA cm^−2^ at 1.23 V (vs. RHE, all the potentials aftermentioned were referenced to RHE if not pointed out) for 4-layered PAD hematite photoanodes. When the deposition layers came to 5 layers, the photocurrent density nearly keeps the same as that of 4-layered photoanode. We note the optimum deposition layers might be 4 layers, with film thickness of about 125 nm [4], which can provide the sufficient light absorption thickness and meanwhile avoid the possible charge recombination caused by too thick films. In this regard, we chose the 4-layered PAD films to study the gradient Ti-doping effect in this work.

**Figure 4:**
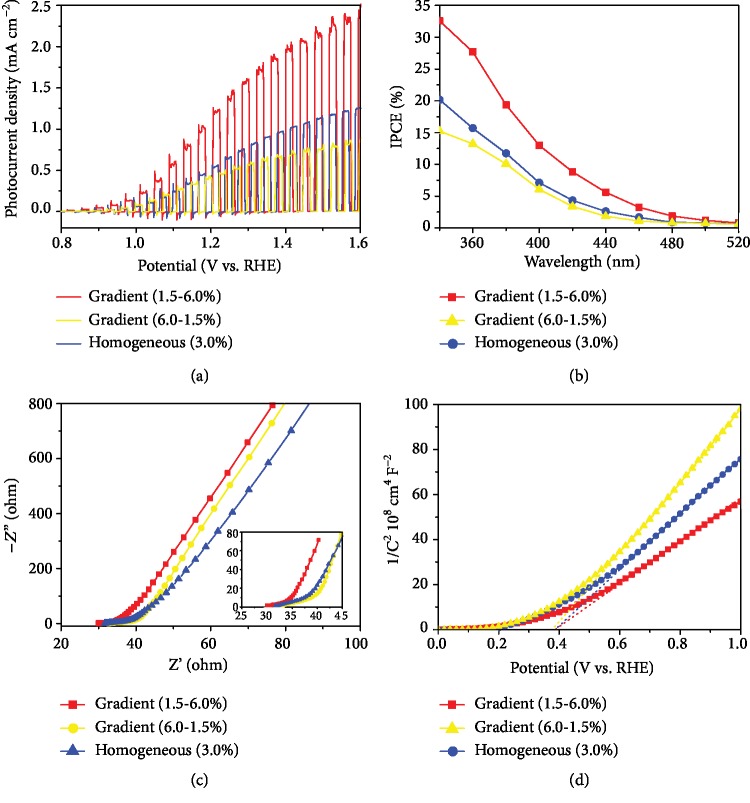
(a) Photocurrent density vs. bias voltage (V vs. RHE) curves and (b) wavelength dependence of IPCE measurements at 1.23 V vs. RHE for hematite photoanodes with homogeneously doped, gradient 1.5-6.0%-doped, and gradient 6.0-1.5%-doped Ti element. The plots were measured in the 1.0 M NaOH aqueous solution under AM 1.5G simulated sunlight. The light was illuminated from the backside of the electrodes. (c) Electrochemical impedance spectra and (d) Mott-Schottky plots of the above different photoelectrodes.

We fabricated typical photoanodes of gradient Ti-doping hematite with 1.5-6.0% and 6.0-1.5% doping level from the surface to the substrate along with the homogenous 3.0% Ti-doped hematite for comparison. Notably, all the photoanode films were deposited with 4-layered thickness. In Figure 4(a), a drastic improvement of PEC water oxidation performance occurs, where the optimal gradient-doped hematite sample (1.5-6.0%) obtains the photocurrent density of about 1.30 mA cm^−2^ at 1.23 V, which is over 2 times higher than that of homogenous-doped hematite (0.55 mA cm^−2^), whereas the gradient-doped hematite (6.0-1.5%) achieves the photocurrent density of 0.47 mA cm^−2^ at 1.23 V. In the meanwhile, we also reproduced several individual gradient samples (1.5-6.0%) shown in [Supplementary-material supplementary-material-1], which all exhibit better PEC performance than the controls. For comparison, the homogeneously doped hematite photoanodes of 4-layered thickness with a different doping level were also fabricated and evaluated by LSV curve ([Supplementary-material supplementary-material-1]), the 3.75% Ti-doped sample of which displays the best PEC performance, achieving a photocurrent density of 0.73 mA cm^−2^ at 1.23 V. We also note that all our fabricated homogenous-doped samples show inferior PEC performance than the gradient-doped hematite (1.5-6.0%). In the meanwhile, the homogeneous 1.5% Ti-doped sample, which features the same surface Ti loading as the gradient-doped sample (1.5-6.0%), exhibits the decreased photocurrent density than the gradient one, also indicating the important role of gradient doping-induced built-in electric filed. It is mentionable that all the doped hematite photoanodes exhibit higher PEC performances than that of the pristine-undoped hematite (0.18 mA cm^−2^ at 1.23 V).

To quantitatively investigate the photoactivity of different doped hematite photoanodes as a function of the wavelength, the incident photon to current efficient (IPCE) measurements was conducted under the bias of 1.23 V. In Figure 4(b), the gradient-doped hematite (1.5-6.0%) photoanode shows an obvious IPCE improvement for all photon wavelengths as compared to the controls (e.g., IPCE of gradient 1.5-6.0%, homogeneous 3.0%, and gradient 6.0-1.5% are 27.5, 16.2, and 13.7% at 360 nm, respectively). We attribute the enhanced IPCE value for two reasons: one is that the built-in electric field would facilitate the charge separation; another is that low-content Ti^4+^ heteroatoms doping on the surface would not destroy the symmetric structure, which would also be beneficial to charge separation and transportation.

### 2.4. Mechanism Analysis of Performance Enhancement

To further shed light on the reasons of the PEC performance improvement, we conducted series of characterization and test. In [Supplementary-material supplementary-material-1], the ultraviolet-visible (UV-Vis) light absorption curves show that there is no obvious difference in the capability of light absorption between the gradient 1.5-6.0%-doped and homogeneous 3.0%-doped hematite photoanodes. The spectrum of the annealing hematite exhibits slightly red-shift, which suggests the slightly enhanced light absorption ability after annealing activation treatment. The optical bandgaps (*E*_g_) were further extrapolated by the following equation: *αhʋ* = *A* (*hʋ* − *E*_g_)^*n*^, where *h* is Planck's constant, *ʋ* is the light frequency, *A* is a constant, and *n* is fixed at 1/2 for an indirect bandgap semiconductor. In [Supplementary-material supplementary-material-1], the fitting *E*_g_ value of the gradient 1.5-6.0%-doped and homogeneous 3.0%-doped hematite photoanodes nearly keep identical (around 2.04 eV), which is also coincided to the reported references [30], whereas the *E*_g_ value of the gradient photoanode after annealing is slightly decreased. Based on the above results, we note that PEC performance improvement is not resulted from the change in light-harvesting properties.

The electrochemical impendency spectroscopy (EIS) was carried out to further study the interfacial properties between the photoanode and the electrolyte. In Figure 4(c), the gradient-doped photoanode (6.0-1.5%) exhibits a slightly larger arches with larger charge-transfer resistance (*R*_ct_) as compared with the gradient doped (1.5-6.0%), which indicates the deficiency of charge transport properties for the 6.0-1.5%-doped photoanodes [31]. In addition, the inset of Figure 4(c) also displays the series resistance (*R*_s_) values of 30.1, 31.7, and 32.7 *Ω* for gradient 1.5-6.0%-doped, homogeneous 3.0%-doped, and gradient 6.0-1.5%-doped photoanodes. Considering the fabrication methods of PAD are similar for all the three samples, we speculate that the built-in electric field would be effective to boost charge transfer ability for the gradient 1.5-6.0%-doped photoanode.

**Figure 5:**
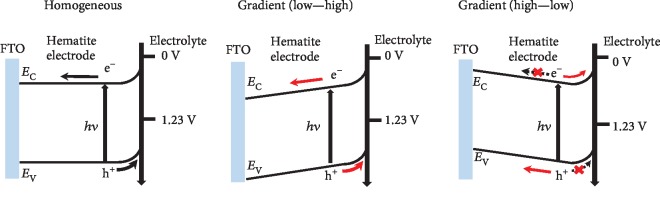
Schematic illustration of the charge transfer in homogeneous- and gradient-doped hematite photoanodes.

To evaluate the flat-band potential (*E*_fb_) and the charge-carrier density (*N*_*d*_) for hematite photoanodes, Mott-Schottky plots were performed based on the capacitances derived from the electrochemical impedance. In Figure 4(d), the calculated *E*_fb_ values for the gradient and homogeneously doped films are all around 0.4 V, which are consistent with the literature values for hematite [17]. In the meanwhile, the *N*_*d*_ values of gradient 1.5-6.0%-doped, homogenous 3.0%-doped, and gradient 6.0-1.5%-doped photoanodes based on the slopes were calculated for 18.88 × 10^19^, 14.22 × 10^19^, and 11.03 × 10^19^ cm^−3^, respectively, clearly showing the decline tendency, which also demonstrates the positive effect of the gradient doping-induced electric field for boosting charge separation and transport. Based on these results, we propose the possible promotion mechanism as shown in Figure 5. For the homogenous-doped hematite photoanode, the charge transport mechanism is strongly dependent on the depletion layer and driven by the charge carrier diffusion; for the gradient-doped hematite photoanode (1.5-6.0%), the built-in electric field pointing to the surface would force the charge separation and result in the smooth arrival of holes on the surface to generate oxygen. Adversely, the gradient-doped films (6.0-1.5%) might provide the opposite electric field, which would hinder the charge transport, further lowing the overall PEC water oxidation performance.

## 3. Discussion

In sum, we developed a facile and controllable layer-by-layer polymer-assisted deposition method by which we precisely modulated the dopant distribution to construct the gradient-doped functional metal oxide films. This directly functionalizes the films with intentional built-in electric field, which can effectively facilitate the charge transport ability. The fabricated gradient-doped hematite photoanode delivers a drastically enhanced photocurrent density of about 1.30 mA cm^−2^ at 1.23 V vs. RHE, which is nearly 100% higher than that of the homogeneously doped hematite photoanode. We highlight that this work will provide a simple and effective strategy to boost the performance of hematite photoanodes, which may further manipulate other earth-abundant transition metal-based photoelectrodes for photochemical water splitting.

## 4. Materials and Methods

### 4.1. Preparation of the PAD Hematite Films

2.20 g Fe(NO_3_)_3_·9H_2_O, 1.40 g EDTA, and 0.80 g PEI (average Mw of ~800) were dissolved in 10 mL DI water, which was then sufficiently stirred to form Fe solution. Afterwards, the ultrafiltration cup was used to remove the uncoordinated small molecules (Fe^3+^ ions, water, and complex molecule) to further obtain the coordinated Fe precursors. Likewise, Ti precursors were prepared using 0.5 mL TiCl_4_ (1.0 M) solutions and 0.8 mL 30 wt% H_2_O_2_ instead of Fe(NO_3_)_3_·9H_2_O, with other procedures keeping unchanged. The contents of Fe and Ti elements in each precursor were determined by inductively coupled plasma atomic emission spectroscopy (ICP-AES), and different doping-level precursors mixed the calculated Fe and Ti precursors. All the PAD films were fabricated with precursors spin coated onto the fluorine-doped tin oxide (FTO) glass substrates, which were then annealed under the temperature of 500°C to remove the organics. The repetitive spin coating and annealing steps were conducted to obtain different-thickness films. To fabricate the gradient-doped hematite films (1.5-6.0% from the surface to the substrate), different doping-level precursors of 6.0, 4.5, 3.0, and 1.5 wt% Ti precursors were step-by-step deposited onto the FTO substrate. To fabricate the gradient-doped hematite films (6.0-1.5% from the surface to the substrate), different doping-level precursors of 1.5, 3.0, 4.5, and 6.0 wt% Ti precursors were step-by-step deposited onto the FTO substrate. The homogeneously doped films were fabricated with 1.5, 3.0, 3.75, and 6.0 wt% Ti precursors deposited and annealed for 4 times, respectively. It is mentionable that all the doped films were annealed under 800°C to activate the PEC performance before the photoelectrochemical test if not mentioned.

### 4.2. Structural Characterizations

The crystal structure of the samples was examined by X-ray diffraction (XRD, D/max2550V). The morphology of the samples was determined by scanning electron microscopy (Hitachi S4800). The crystal structure was determined by micro-Raman spectroscopy (Renishaw, inVia Reflex). Chemical valence states were analyzed by X-ray photoelectron spectroscopy (XPS, Thermo Escalab 250) with Al K*α* X-ray beam (1486.6 eV), and all binding energies were calibrated using the C 1s peak at 284.8 eV as the reference. The optical absorption spectra of the samples were recorded in a UV/Vis spectrophotometer. The Fe and Ti contents in the precursor solution were analyzed by inductively coupled plasma atomic emission spectroscopy (ICP-AES, Vanan 710). The Fe and Ti contents of the PAD films in the different depth were characterized by time-of-flight secondary ion mass spectrometry (ToF-SIMS, AXIMA QTT, Shimadzu). XAFS spectra at the Fe K-edge were performed on the 1W1B beamline station of the Beijing Synchrotron Radiation Facility (BSRF), China.

### 4.3. PEC Analysis

All linear sweep voltammograms (LSV) were measured by a CHI 660e electrochemical workstation in a three-electrode configuration using a Pt foil counter electrode and an Ag/AgCl (3.5 M KCl) reference electrode. An aqueous solution of NaOH (1.0 M) was utilized as the electrolyte, and LSV curves were scanned from -500 to 800 mV vs. Ag/AgCl. The LSV curve of the gradient photoanode (1.5-6.0%) shown in this work was chosen from 6 individual samples with the optimal performance, and the average performance with error bars was taken from these 6 individual samples. The measurements were performed under one sun condition using a solar light simulator (Oriel, 91160, AM 1.5 globe). The power of the simulated light was adjusted to 100 mW cm^−2^.

### 4.4. Electrochemical Measurements

The EIS experiments and Mott-Schottky plots were measured in the dark by using an electrochemical workstation (Parstat 2273, Princeton). The frequency range of EIS experiments was from 100 kHz to 100 mHz. The Mott-Schottky calculations were derived from impedance measurements in the dark sweeping from -1.0 to 0 V vs. Ag/AgCl with 10 mV increments. The AC potential frequency was 3000 Hz with the amplitude of 10 mV. The charge-carrier density (*N*_*d*_) for the abovementioned hematite photoanodes was calculated with the following equation:

(1) $$\begin{array}{c} N_{d}=\left(\frac{2}{e{\varepsilon \varepsilon }_{o}}\right){\left[\frac{d\left(1/C^{2}\right)}{dV}\right]}^{-1}, \end{array}$$

where *e* = 1.60 × 10^−19^ C is the electron charge, *ε* = 80 is the dielectric constant of hematite, *ε*_*o*_ = 8.85 × 10^−14^ F cm^−1^ is the vacuum permittivity, *C* is the capacitance of the space charge region, and *V* is the electrode applied potential.

## Supplementary Materials

Supplementary Materials Figure S1: XRD patterns of different PAD photoelectrodes. Figure S2: XPS spectra of surface 1.5% Ti-doped hematite photoelectrode. Figure S3: the enlarged XANES spectra of the PAD hematite films with different Ti-doping levels. Figure S4: PEC measurements of hematite photoelectrodes composed of 1-5 PAD layers. Figure S5: photocurrent density vs. bias voltage (V vs. RHE) curves of several individual devices of gradient-doped hematites with a doping level from 1.5 to 6.0%. Figure S6: PEC performance of hematite photoanodes with different homogeneous doping level. Figure S7: UV-Vis spectra and Tauc plots of different PAD electrodes.

## References

[1] Walter M. G., Warren E. L., McKone J. R. (2010). Solar water splitting cells. *Chemical Reviews*.

[2] Ang E. H., Dinh K. N., Sun X. (2019). Highly efficient and stable hydrogen production in all pH range by two-dimensional structured metal-doped tungsten semicarbides. *Research*.

[3] Kim J. H., Hansora D., Sharma P., Jang J. W., Lee J. S. (2019). Toward practical solar hydrogen production – an artificial photosynthetic leaf-to-farm challenge. *Chemical Society Reviews*.

[4] Sivula K., Le Formal F., Grätzel M. (2011). Solar water splitting: progress using hematite (*α*-Fe_2_O_3_) photoelectrodes. *ChemSusChem*.

[5] Li M., Luo W., Cao D. (2013). A co-catalyst-loaded Ta_3_N_5_ photoanode with a high solar photocurrent for water splitting upon facile removal of the surface layer. *Angewandte Chemie International Edition*.

[6] Li W., Da P., Zhang Y. (2014). WO_3_ nanoflakes for enhanced photoelectrochemical conversion. *ACS Nano*.

[7] Sambur J. B., Chen T. Y., Choudhary E. (2016). Sub-particle reaction and photocurrent mapping to optimize catalyst-modified photoanodes. *Nature*.

[8] Puthirath Balan A., Radhakrishnan S., Woellner C. F. (2018). Exfoliation of a non-van der Waals material from iron ore hematite. *Nature Nanotechnology*.

[9] Pan L., Kim J. H., Mayer M. T. (2018). Boosting the performance of Cu_2_O photocathodes for unassisted solar water splitting devices. *Nature Catalysis*.

[10] Zhang K., Jin B., Park C. (2019). Black phosphorene as a hole extraction layer boosting solar water splitting of oxygen evolution catalysts. *Nature Communications*.

[11] Fang T., Huang H., Feng J. (2019). Reactive inorganic vapor deposition of perovskite oxynitride films for solar energy conversion. *Research*.

[12] Tilley S. D., Cornuz M., Sivula K., Grätzel M. (2010). Light-induced water splitting with hematite: improved nanostructure and iridium oxide catalysis. *Angewandte Chemie International Edition*.

[13] Cesar I., Sivula K., Kay A., Zboril R., Grätzel M. (2009). Influence of feature size, film thickness, and silicon doping on the performance of nanostructured hematite photoanodes for solar water splitting. *The Journal of Physical Chemistry C*.

[14] Monllor-Satoca D., Bärtsch M., Fàbrega C. (2015). What do you do, titanium? Insight into the role of titanium oxide as a water oxidation promoter in hematite-based photoanodes. *Energy & Environmental Science*.

[15] Wang T., Luo W., Wen X., Zou Z., Huang W. (2016). Nonequilibrium Ti^4+^ Doping significantly enhances the performance of Fe_2_O_3_ Photoanodes by quenching. *ChemNanoMat*.

[16] Subramanian A., Gracia-Espino E., Annamalai A. (2018). Effect of tetravalent dopants on hematite nanostructure for enhanced photoelectrochemical water splitting. *Applied Surface Science*.

[17] Wang C. W., Yang S., Fang W. Q., Liu P., Zhao H., Yang H. G. (2016). Engineered hematite mesoporous single crystals drive drastic enhancement in solar water splitting. *Nano Letters*.

[18] Xi L., Chiam S. Y., Mak W. F. (2013). A novel strategy for surface treatment on hematite photoanode for efficient water oxidation. *Chemical Science*.

[19] Feng C., Fu S., Wang W., Zhang Y., Bi Y. (2019). High-crystalline and high-aspect-ratio hematite nanotube photoanode for efficient solar water splitting. *Applied Catalysis B: Environmental*.

[20] He H., Liao A., Guo W., Luo W., Zhou Y., Zou Z. (2019). State-of-the-art progress in the use of ternary metal oxides as photoelectrode materials for water splitting and organic synthesis. *Nano Today*.

[21] Yu Y., Yan W., Wang X. (2018). Surface engineering for extremely enhanced charge separation and photocatalytic hydrogen evolution on g-C_3_N_4_. *Advanced Materials*.

[22] Qiao H. W., Yang S., Wang Y. (2019). A gradient heterostructure based on tolerance factor in high-performance perovskite solar cells with 0.84 fill factor. *Advanced Materials*.

[23] Abdi F. F., Han L., Smets A. H. M., Zeman M., Dam B., van de Krol R. (2013). Efficient solar water splitting by enhanced charge separation in a bismuth vanadate-silicon tandem photoelectrode. *Nature Communications*.

[24] Luo Z., Li C., Liu S., Wang T., Gong J. (2017). Gradient doping of phosphorus in Fe_2_O_3_ nanoarray photoanodes for enhanced charge separation. *Chemical Science*.

[25] Yu Y., Huang Y., Yu Y., Shi Y., Zhang B. (2018). Design of continuous built-in band bending in self-supported CdS nanorod-based hierarchical architecture for efficient photoelectrochemical hydrogen production. *Nano Energy*.

[26] Ahn H. J., Goswami A., Riboni F. (2018). Hematite photoanode with complex nanoarchitecture providing tunable gradient doping and low onset potential for photoelectrochemical water splitting. *ChemSusChem*.

[27] Zou G. F., Zhao J., Luo H. M., McCleskey T. M., Burrell A. K., Jia Q. X. (2013). Polymer-assisted-deposition: a chemical solution route for a wide range of materials. *Chemical Society Reviews*.

[28] Magnan H., Stanescu D., Rioult M., Fonda E., Barbier A. (2012). Enhanced photoanode properties of epitaxial Ti doped *α*-Fe_2_O_3_ (0001) thin films. *Applied Physics Letters*.

[29] Liu P. F., Yang S., Zhang B., Yang H. G. (2016). Defect-rich ultrathin cobalt–iron layered double hydroxide for electrochemical overall water splitting. *ACS Applied Materials & Interfaces*.

[30] Jang J. W., Du C., Ye Y. (2015). Enabling unassisted solar water splitting by iron oxide and silicon. *Nature Communications*.

[31] Wang C. W., Yang S., Jiang H. B., Yang H. (2015). Chemical vapor deposition of FeOCl nanosheet arrays and their conversion to porous *α*-Fe_2_O_3_ Photoanodes for photoelectrochemical water splitting. *Chemistry – A European Journal*.

