# Supplementary material for: Ultrathin Hematite Photoanode with Gradient Ti Doping
Source: Research (Wash D C). 2020 Feb 24;2020:5473217. doi: 10.34133/2020/5473217 (PMC7060458; doi:10.34133/2020/5473217)
Supplement: Supplementary Materials — Figure S1: XRD patterns of different PAD photoelectrodes. Figure S2: XPS spectra of surface 1.5% Ti-doped hematite photoelectrode. Figure S3: the enlarged XANES spectra of the PAD hematite films with different Ti-doping levels. Figure S4: PEC measurements of hematite photoelectrodes composed of 1-5 PAD layers. Figure S5: photocurrent density vs. bias voltage (V vs. RHE) curves of several individual devices of gradient-doped hematites with a doping level from 1.5 to 6.0%. Figure S6: PEC performance of hematite photoanodes with different homogeneous doping level. Figure S7: UV-Vis spectra and Tauc plots of different PAD electrodes. [file 5473217.f1.doc]

Supporting Information

Ultrathin Hematite Photoanode with Gradient Ti Doping

Pengfei Liu^1^†, Chongwu Wang^2^†, Lijie Wang^1^, Xuefeng Wu^1^, Lirong Zheng^3^, and Hua Gui Yang^1^*

^1^ Key Laboratory for Ultrafine Materials of Ministry of Education, Shanghai Engineering Research Center of Hierarchical Nanomaterials, School of Materials Science and Engineering, East China University of Science and Technology, 130 Meilong Road, Shanghai 200237, China.

^2^ School of Electrical and Electronic Engineering, Nanyang Technological University, 50 Nanyang Avenue, Singapore 639798, Singapore.

^3^ Institute of High Energy Physics, Chinese Academy of Sciences, Beijing 100049, China.

Correspondence should be addressed to Hua Gui Yang; hgyang@ecust.edu.cn.

† These authors contributed equally to this work.

**Chemicals:** Iron(III) nitrate nonahydrate (Fe(NO_3_)_3_·9H_2_O) and ethylenediaminetetraacetic acid (EDTA, C_10_H_16_N_2_O_8_) were purchased from Sinopharm Chemical Reagent Co. Ltd. Titanium tetrachloride (TiCl_4_) and polyethylenimine (PEI) were bought from Sigma-Aldrich. Deionized (DI) water used in our experiments was supplied by Milli-Q System (Millipore, Billerica, MA).


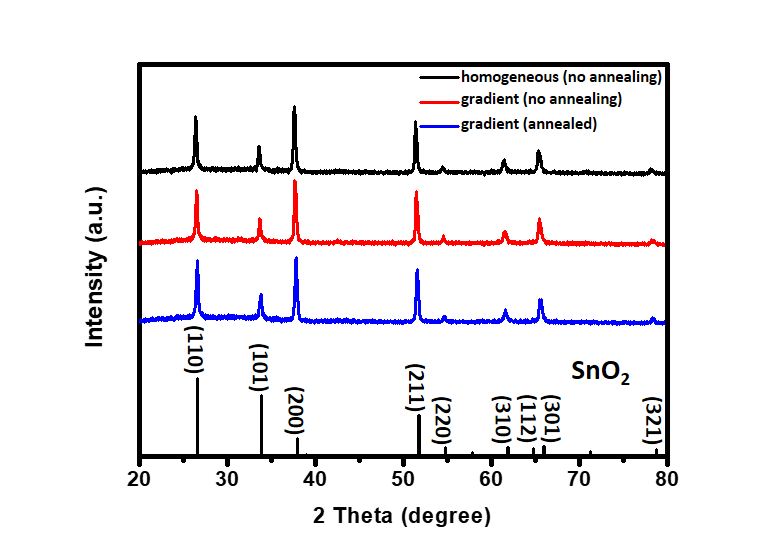


**Figure S1.** XRD patterns of homogeneously doped, gradient doped and annealed PAD electrodes. No peaks of hematite can be observed.


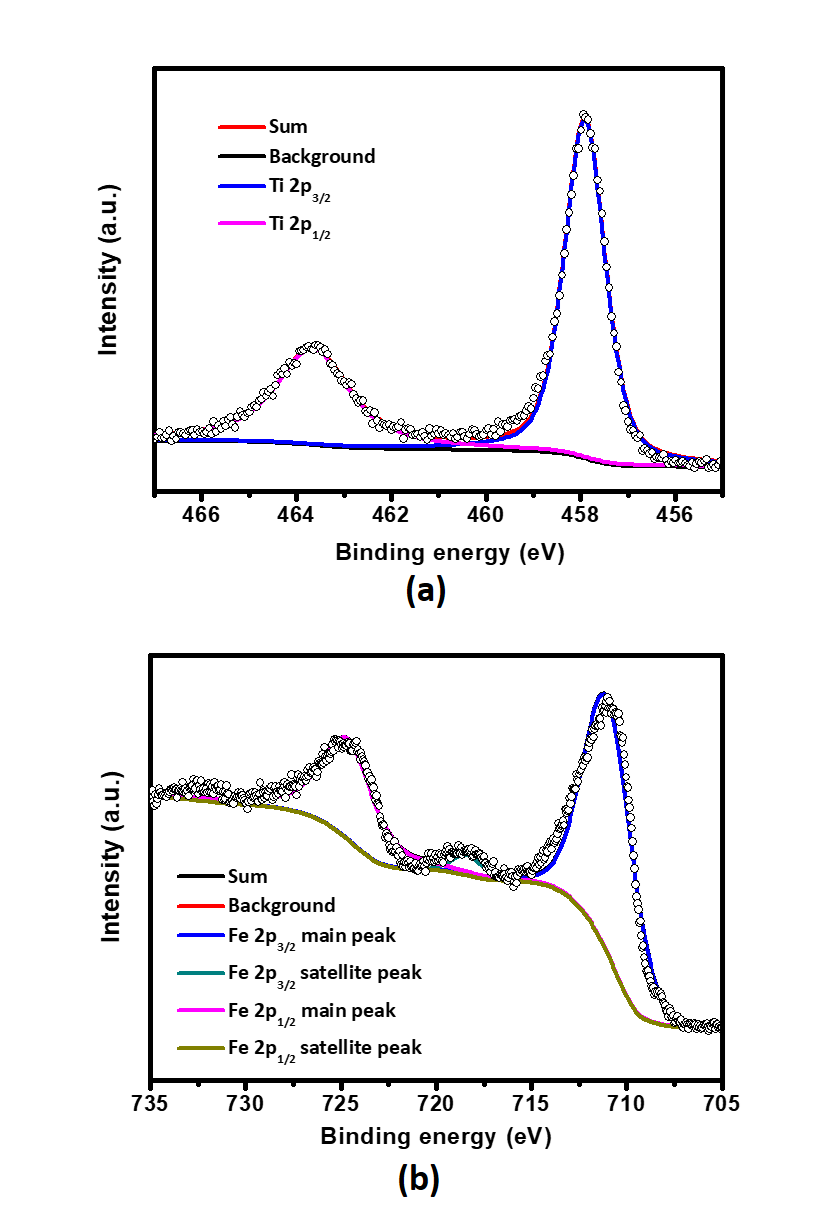


**Figure S2.** XPS spectra of 1.5% Ti doped hematite photoelectrode of (a) Ti 2p region illustrating the existence Ti^4+^ and (b) Fe 2p region indicating that Fe element is in the form of Fe^3+^.


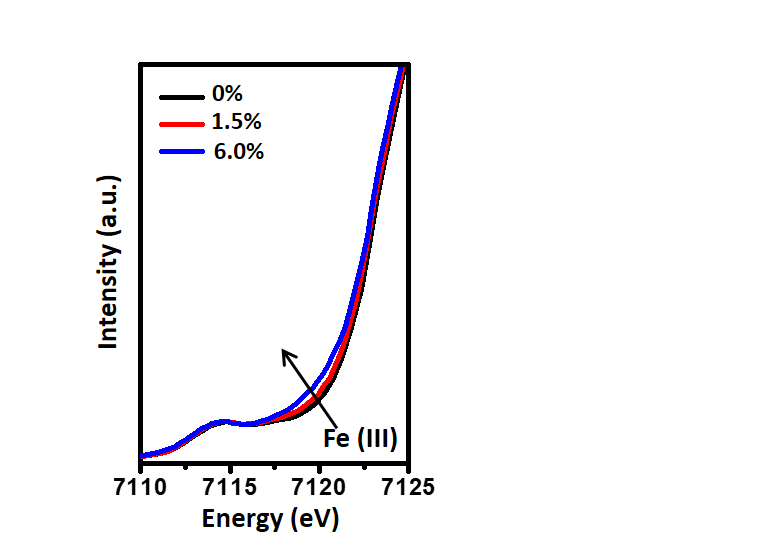


**Figure S3.** The enlarged XANES spectra of the PAD hematite films with different Ti doping levels.


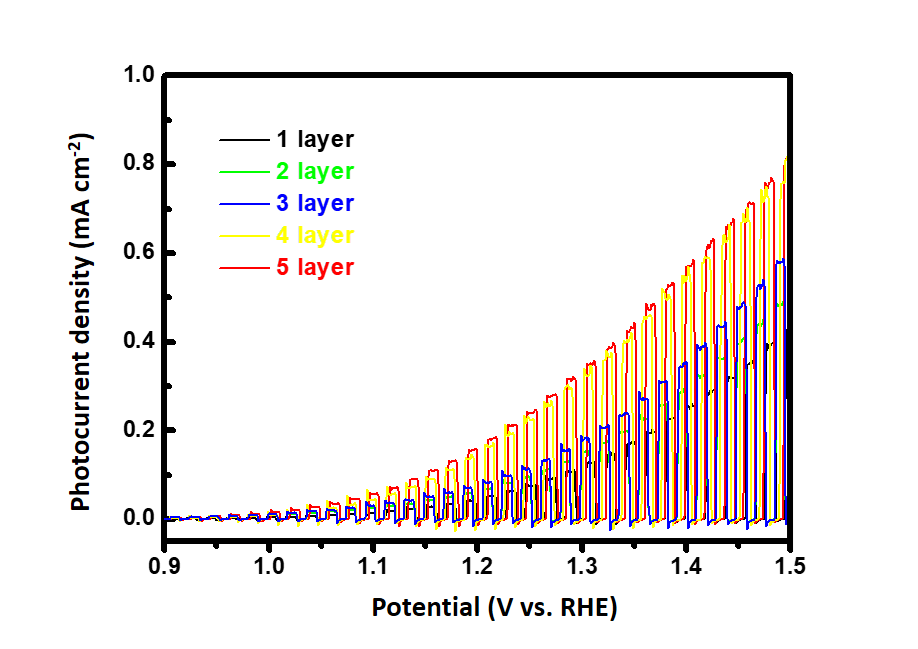


**Figure S4.** PEC measurements of hematite photoelectrodes composed of 1-5 PAD layers.


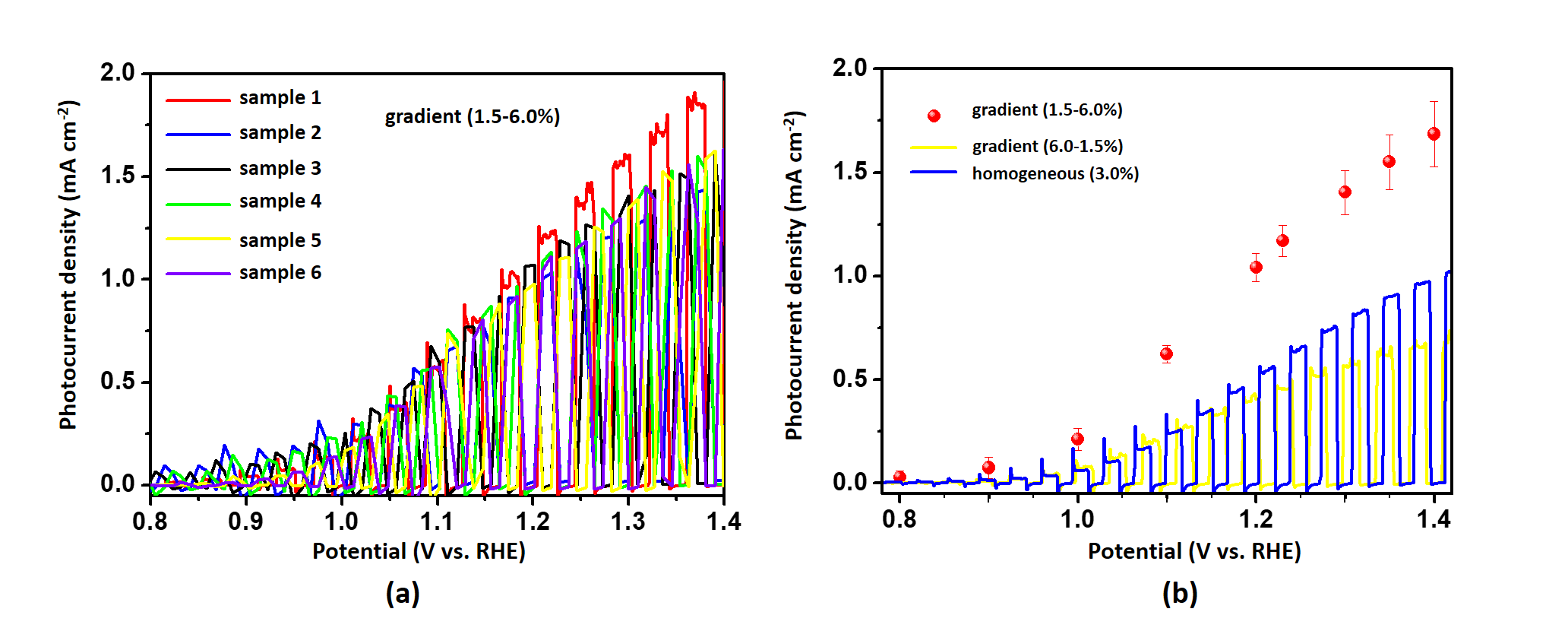


**Figure S5.** Photocurrent density vs. bias voltage (V vs. RHE) curves of (a) individual devices of gradient doped hematites with doping level from 1.5 to 6.0% and (b) gradient doped samples (1.5-6.0%) with error bars based on 6 individual devices from (a) and controlld samples.


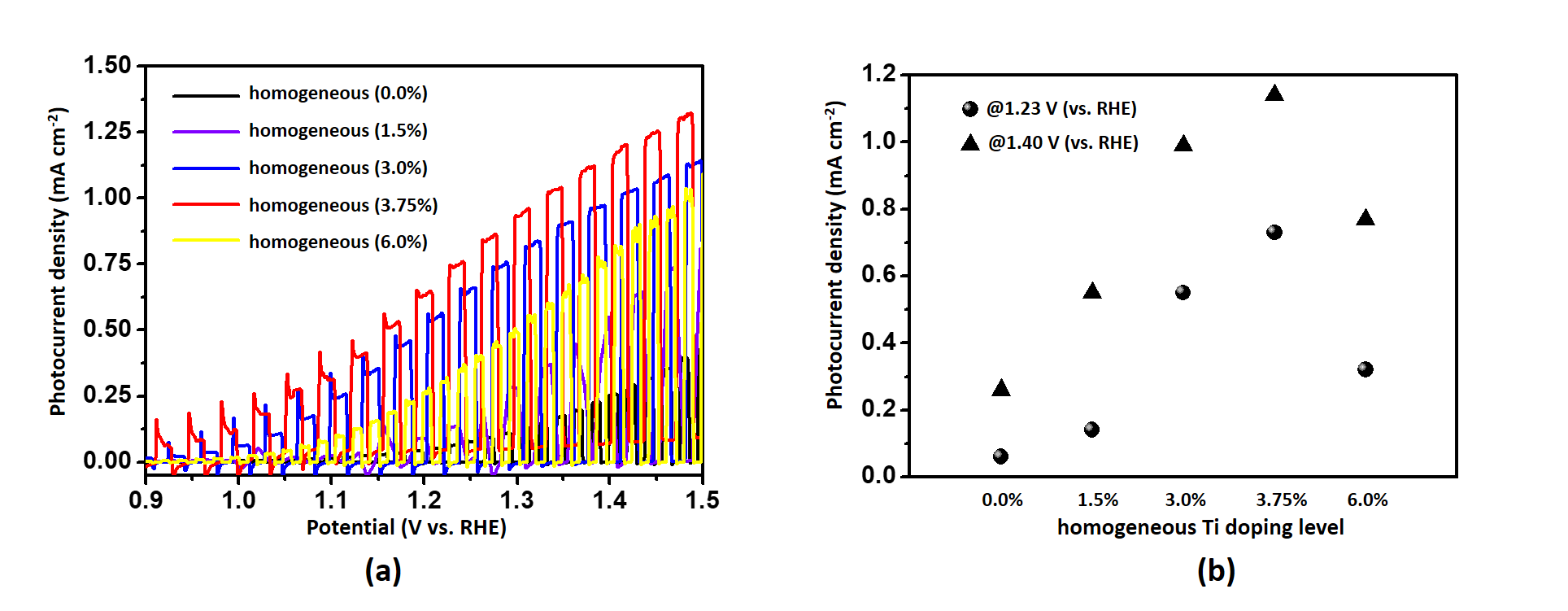


**Figure S6.** (a) Photocurrent density vs. bias voltage (V vs. RHE) curves of homogeneously doped hematite photoanodes with different Ti doping levels. (b) The typical photocurrent densities for homogeneously doped hematite photoanodes with different Ti doping levels at the bias voltages of 1.23 and 1.40 V, respectively.


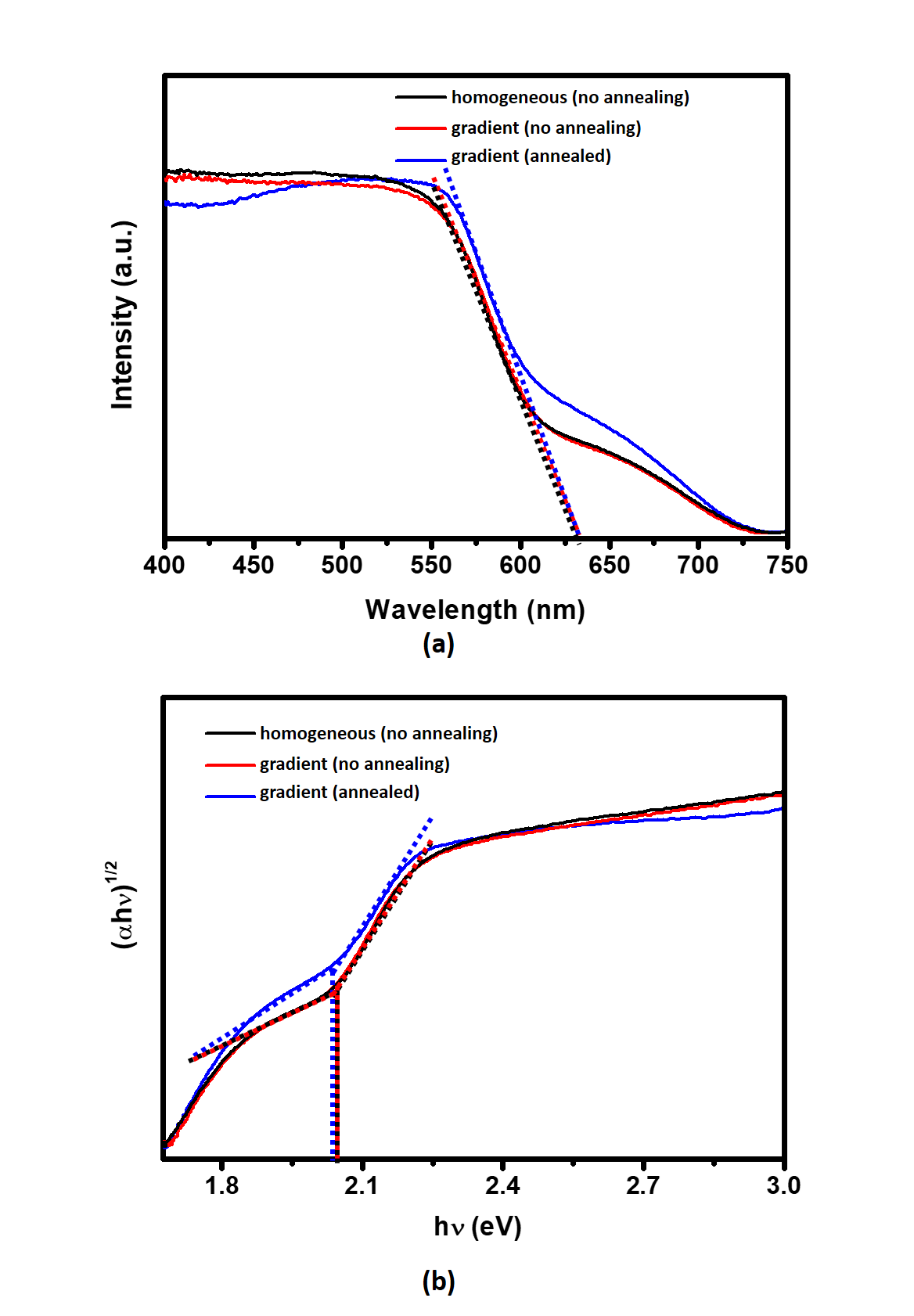


**Figure S7.** (a) UV-Vis absorption spectra and (b) Tauc plots of homogeneously doped, gradient doped and annealed gradient doped hematite photoelectrode.
